# Production, Mechanical and Functional Properties of Long-Length TiNiHf Rods with High-Temperature Shape Memory Effect

**DOI:** 10.3390/ma16020615

**Published:** 2023-01-09

**Authors:** Roman Karelin, Victor Komarov, Vladimir Cherkasov, Vladimir Yusupov, Sergey Prokoshkin, Vladimir Andreev

**Affiliations:** 1 Baikov Institute of Metallurgy and Materials Science RAS, Moscow 119334, Russia; 2 National University of Science and Technology MISIS, Moscow 119049, Russia

**Keywords:** shape memory alloys, rotary forging, structure, mechanical properties, NiTiHf

## Abstract

In the present work, the possibility of manufacturing long-length TiNiHf rods with a lowered Hf content and a high-temperature shape memory effect in the range of 120–160 °C was studied. Initial ingots with 1.5, 3.0 and 5.0 at.% Hf were obtained by electron beam melting in a copper water-cooled stream-type mold. The obtained ingots were rotary forged at the temperature of 950 °C, with the relative strain from 5 to 10% per one pass. The obtained results revealed that the ingots with 3.0 and 5.0 at.% Hf demonstrated insufficient technological plasticity, presumably because of the excess precipitation of (Ti,Hf)_2_Ni-type particles. The premature destruction of ingots during the deformation process does not allow obtaining high-quality long-length rods. A long-length rod with a diameter of 3.5 mm and a length of 870 mm was produced by rotary forging from the ingot with 1.5 at.% Hf. The obtained TiNiHf rod had relatively high values of mechanical properties (a dislocation yield stress σ_y_ of 800 MPa, ultimate tensile strength σ_B_ of 1000 MPa, and elongation to fracture δ of 24%), functional properties (a completely recoverable strain of 5%), and a required finishing temperature of shape recovery of 125 °C in the as-forged state and of 155 °C after post-deformation annealing at 550 °C for 2 h.

## 1. Introduction

Ti-Ni-based shape memory alloys (SMAs) are functional materials, actively used for the manufacturing of various devices and construction elements for engineering or medical applications [1,2,3,4,5]. Today, permanent technological development leads to the formation of new special requirements for the operational characteristics of applied alloys [6,7,8,9,10,11]. For example, great attention and high demand are observed for Ti-Ni-based alloys with a high-temperature shape memory effect.

Ternary high-temperature Ti-Ni-based SMAs (TiNiX), where X is a precious metal, such as Pd, Pt, etc., or where X is Zr or Hf, are actively developed and studied [12,13,14,15]. However, the first group of alloys are too expensive due to the use of precious metals as alloying elements, and the second group exhibits insufficient deformability. TiNiHf alloys seem to be more attractive for wide practical application because they perform required mechanical and functional properties in combination with relatively low cost and greater stability of the operational characteristics as compared to TiNiZr alloys [15]. Therefore, to date, numerous studies of TiNiHf alloys revealed the main features of the formation of their structural state and properties [16,17,18,19,20,21,22,23,24,25,26,27,28,29,30,31,32,33,34]. Ternary TiNiHf alloys are usually divided into two groups, one with increased Ni content and one with reduced Ni content, similarly to binary TiNi SMAs. According to studies [16,17,18,19,20], alloys with low Ni content (less than 49.5 at.%) have insufficient technological plasticity due to the precipitation of the (Ti, Hf)_2_Ni-type phase. An increase in the Ni content leads to a decrease in the characteristic temperatures of martensitic transformations; therefore, in order to obtain a high-temperature state in the alloy, the content of Hf also should be increased [16,21]. That leads to the increase in the production cost and makes it difficult to obtain a temperature range of shape recovery between 100 and 200 °C. For example, the most commonly applied Ti_29_._7_Ni_50_._3_Hf_20_ alloy has the finishing temperature of shape recovery of about 300 °C or higher, in dependence with the applied melting and deformation modes [16,17,19]. Binary TiNi near-equiatomic or Ti-enriched alloys also do not provide such a temperature range of shape recovery. The finishing temperature of the reverse MT in binary alloys hardly exceeds 110 °C. Additionally, in Ni-rich TiNiHf alloys, the aging process is developed during thermal treatment in the temperature range of 400–650 °C [23,24,25,26,27,28]. That allows precisely changing the combination of properties but may affect the functional characteristics of applied devices during the operation at higher temperatures. This factor must be considered during the development of technological schemes for the production of various devices from TiNiHf SMAs. Another problem, as was already mentioned, is the sufficiently low deformability of TiNiHf alloys. Therefore, the problem of obtaining high-quality long-length rods from TiNiHf alloys with low Ni and Hf contents and with a finishing temperature of shape recovery of about 150 °C is unsolved.

The melting processes of TiNi-based SMAs from pure charge components are often associated with some difficulties, such as an increased concentration of gas impurities due to the high reactivity of pure titanium. For TiNiHf alloys, an increase in the concentration of gas impurities, in particular oxygen, is especially critical because of an increase in the concentration of embrittling (Ti, Hf)_2_Ni-type excess phases with a high concentration of Ti and Hf, as well as HfO_2_ in alloys with a high concentration of Hf and Ni [16,17,18,19,20]. Therefore, the application of finished Ti-Ni billets as a charge component for melting TiNiHf alloys may decrease the concentration of gas impurities due to the absence of pure titanium during the melting process. The application of this concept also expands the possibilities of recycling TiNi binary alloys.

Based on the foregoing, the aim of the present work consisted in the production of high-quality long-length rods from TiNiHf alloys with a reduced content of nickel and hafnium, providing high mechanical and functional properties, in combination with a finishing temperature of the reverse martensitic transformation of about 120–160 °C through the application of finished Ti-Ni billets and high-purity hafnium wire during the melting process and various modes of thermomechanical treatment.

## 2. Materials and Methods

A polished TiNi SMA rod with a diameter of 12 mm, manufactured by industrial center MATEK SMA Ltd., Moscow, Russia and cold-worked hafnium wire with a diameter of 2 mm, manufactured by E.A. Yudin Chemical and Metallurgical Plant, Novosibirsk, Russia were chosen as the charge materials for the melting of TiNiHf initial ingots. The chemical composition of the applied TiNi rod and Hf wire is shown in Table 1 and Table 2, respectively.

Initial TiNiHf ingots were obtained by electron beam melting (EBM) in a furnace with a power of 60 kW at a vacuum of 1 × 10^−5^ in a copper water-cooled stream-type crystallizer. The dimensions, weight, and chemical composition of the obtained ingots are shown in Table 3.

After melting, the ingots were homogenized at 1000 °C for 1 h in a vacuum and rotary forged at the temperature of 950 °C, with a relative strain from 5 to 10% per one pass. After RF, post-deformation annealing (PDA) at 550 °C for 2 h was applied. The microstructure was studied using a *UNION* optical microscope and the scanning electron microscope (SEM) Scios (Thermo Fisher Scientific, Waltham, MA, USA) at an accelerating voltage of 10 kV. The investigation of the chemical composition by energy dispersive X-ray spectroscopy (EDS) was carried out using the EDX system from *EDAX*. The study of the temperatures of forward (M_s_, M_f_) and reverse (A_s_, A_f_) martensitic transformations (MTs) was carried out using a Mettler Toledo differential scanning calorimeter in the cooling-heating cycle at a rate of 10 K/min in the temperature range from minus 40 to 200 °C. The phase composition was studied using a Dron-3 X-ray diffractometer in Cu_Kα_ radiation in the 2Θ angle range from 20 to 80° at room temperature. The Vickers hardness measurements were carried out at room temperature using a LECOM 400-A tester under a load of 1 N. The mechanical properties were determined at room temperature by uniaxial tensile tests using the universal tensile machine INSTRON 3382, with a deformation rate of 2 mm/min. The following mechanical parameters were determined: critical stress for martensite reorientation (transformation yield stress) σ_cr_, dislocation yield stress σ_y_, ultimate tensile strength σ_B_, and elongation to failure δ. The measurement of functional properties, such as total completely recoverable strain (Ɛ_rt_) and the temperature range of shape recovery (TRSR), was carried out using the thermomechanical method by deformation in bending. The bending of preliminary cut samples with the size of 0.4 × 0.6 × 20 mm was conducted at room temperature around mandrels with various diameters. The deformation value was determined based on the following relationship: Ɛ = d/(D + d), where d is the diameter of the sample, and D is the diameter of the mandrel. The shape recovery rate (SRR) was determined as a ratio of recovered strain to induced strain, Ɛ_rt_/Ɛ_i_*100% [35]. After the strain induction, the sample was heated in oil to implement the shape memory effect and determine the Ɛ_rt_ and TRSR.

## 3. Results and Discussion

### 3.1. The Features of the Manufacturing Process of Initial Ingots and Long-Length Rods from TiNiHf Alloys

**Figure 1:**
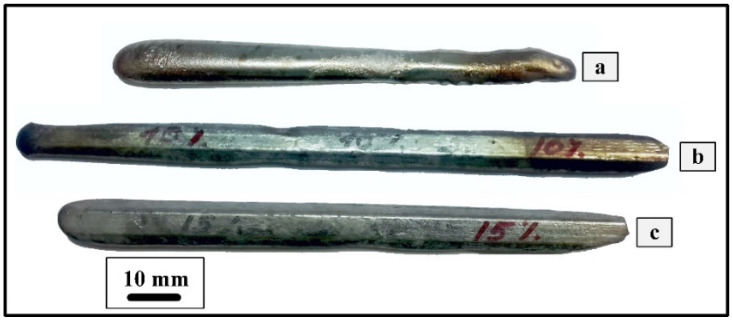
Images of TiNiHf initial ingots obtained by electron beam melting in a copper water-cooled stream-type mold: 1.5 at.% of Hf (**a**), 3.0 at.% of Hf (**b**), and 5.0 at.% of Hf (**c**).

Initial ingots were produced by EBM. EBM has a number of advantages in comparison to induction and electric arc melting, including the effective purification of metals from gas and metallic impurities; the absence of defects of shrinkage origin in ingots because of the possibility of a smooth change in the power of the electron beam and complete filling of the shrinkage cavity; and the possibility of using charge materials in any form. The shape of the obtained ingots in a stream-type mold allows applying subsequent thermomechanical treatment immediately after melting, without additional mechanical operations. Images of the obtained ingots are shown in Figure 1.

**Figure 2:**
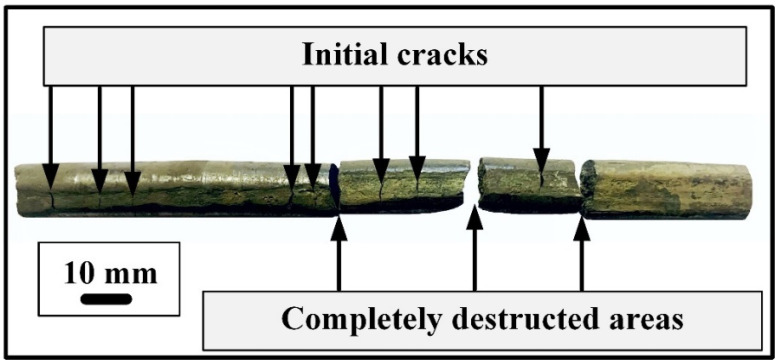
Image of TiNiHf sample with 3.0 at.% Hf after several passes of rotary forging.

Albeit hot rotary forging, the most favorable forming technique for the processing of hard-to-deform metallic materials [36], was applied to produce long-length rods from initial ingots, only the first ingot with 1.5 at.% HF was successfully forged to a diameter of 3.5 mm and a length of 870 mm. Ingots with 3.0 and 5.0 at.% Hf were destructed (Figure 2) during the first few passes because of insufficient technological plasticity, presumably because of the increase in the amount of (Ti,Hf)_2_Ni-type brittle phase with the decrease in Ni content. The premature destruction of ingots during the process of rotary forging does not allow obtaining high-quality long-length rods. Therefore, further investigation of mechanical and functional properties, except the temperature range of martensitic transformations, was carried out only for the TiNiHf rod with 1.5. at.% of Hf.

### 3.2. Microstructural and Phase Analysis

**Figure 3:**
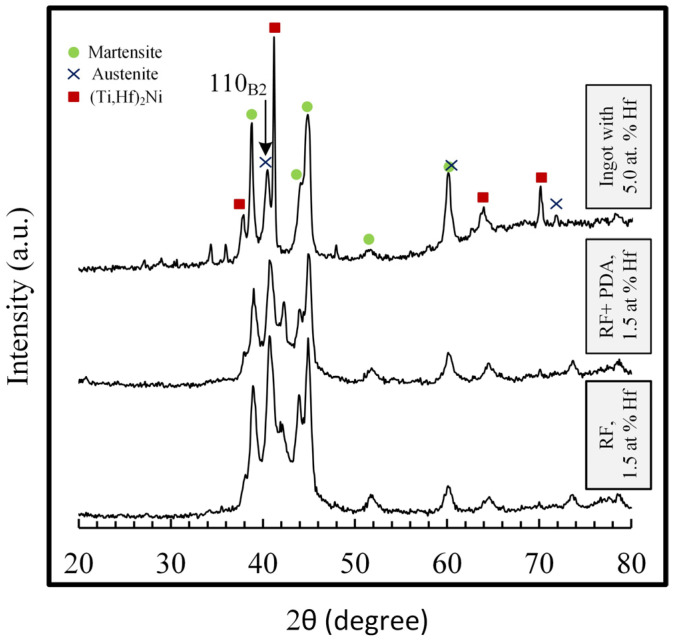
X-ray diffractograms of TiNiHf SMA rods with a diameter of 3.5 mm after RF and RF + PDA 550 °C for 2 h.

The X-ray diffractograms of TiNiHf rods with a diameter of 3.5 mm in the as-forged state and after additional PDA at 550 °C for 2 h are shown in Figure 3.

The obtained results revealed that after rotary forging the studied TiNiHf alloys at room temperature, the main phase was B19′-martensite, and some amount of B2-austenite phase is also presented. X-ray lines corresponding to the (Ti,Hf)_2_Ni-type particles of excess phase were clearly observed. PDA at 550 °C for 2 h led to the narrowing of X-ray line peaks, indicating the decrease in the density of structural defects and accompanied by a slight increase in the proportion of the B2-austenite phase, and some increase in the intensity of the 110_B2_ line can be observed. The phase composition, however, was practically the same as after RF.

**Figure 4:**
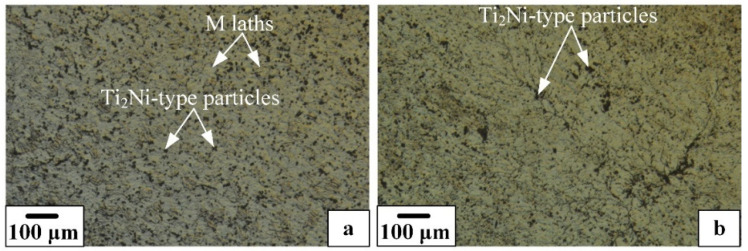
Microstructure of the TiNiHf SMA rods with a diameter of 3.5 mm after RF (**a**) and RF + PDA 550 °C for 2 h (**b**). Light optical microscopy.

Analyses of the microstructure revealed that after rotary forging, a dynamically recrystallized structure with an average grain size in the range of 20–30 microns and a huge number of precipitated (Ti,Hf)_2_Ni-type particles was formed in the sample (Figure 4a). Post-deformation annealing did not lead to changes in the structure, noticeable by light microscopy (Figure 4b).

**Figure 5:**
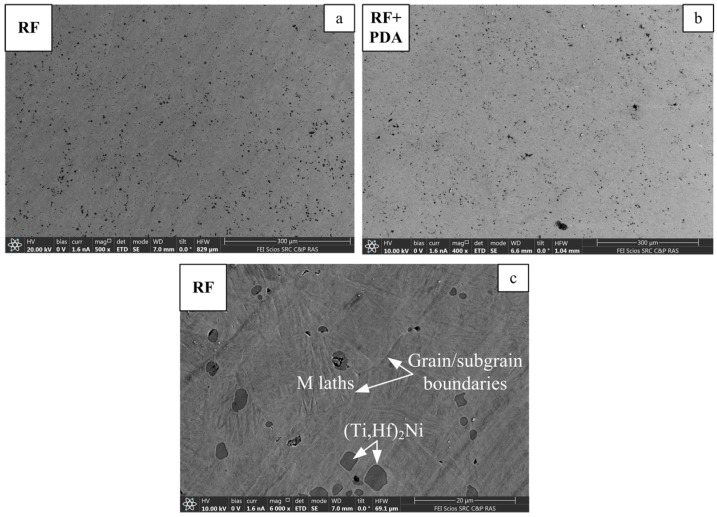
SEM images of TiNiHf rods after RF (**a**,**c**) and RF + PDA (**b**).

**Figure 6:**
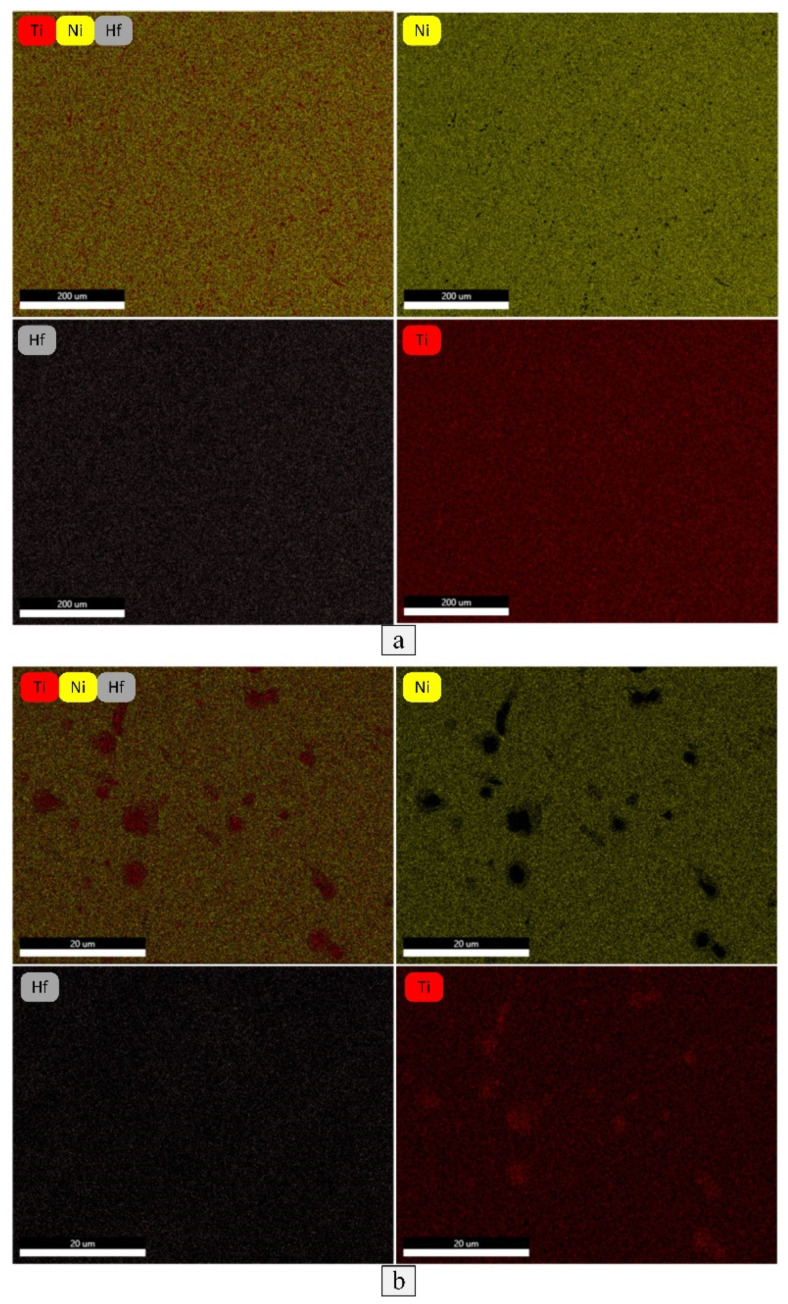
Elemental EDS mapping of Ti, Ni and Hf in TiNiHf rods after RF (**a**) and with higher resolution (**b**).

The SEM images, obtained after RF and RF + PDA, are shown in Figure 5. The black rounded and elongated inclusions are associated with the (Ti,Hf)_2_Ni-type phase based on the results of the EDS analysis (Figure 5). The definition of structural elements and grain/subgrain boundaries is complicated due to the martensitic state of the studied rods at room temperature. The difference in the structural state with the application of PDA was not determined by the SEM, just like after light microscopy. The elemental maps, shown in Figure 6a, indicate the elemental distribution of Ni, Ti and Hf in the obtained rods. Elemental EDS mapping revealed the practically homogeneous distribution of elements, albeit indicating small Ti-rich and Ni-poor areas, presumably corresponding to the (Ti,Hf)_2_Ni-type particles. The elemental maps, taken with higher resolutions in order to study the elemental distribution along the precipitates and their surrounding matrix, confirm this presumption (Figure 6b).

### 3.3. Temperature Ranges of Martensitic Transformations

**Figure 7:**
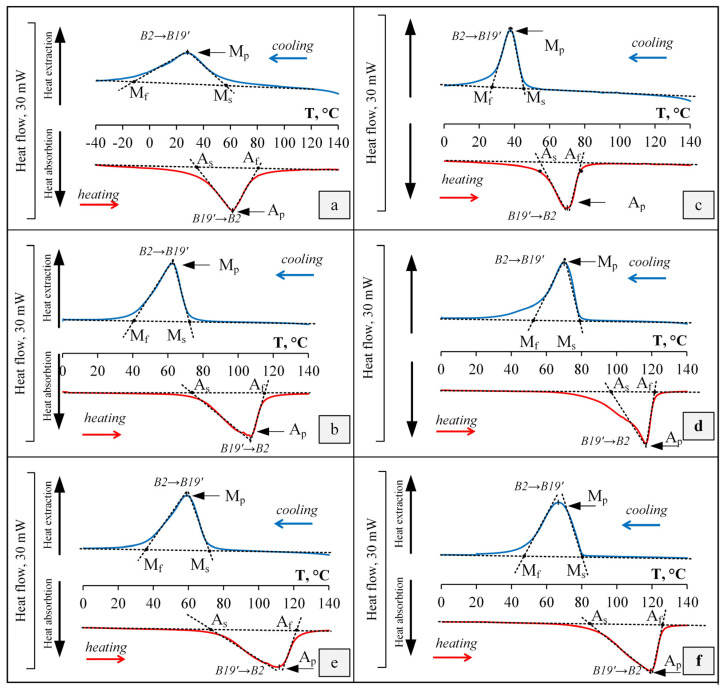
Calorimetric curves of the TiNiHf initial ingots in as-cast state and after annealing at 1000 °C for 1 h: 1.5 at.% Hf—(**a**,**c**), 3.0 at.% Hf—(**b**,**d**), and 5.0 at.% Hf—(**e**,**f**).

The results of differential scanning calorimetry are presented in Figure 7. 

Based on the analysis of the obtained calorimetric curves, summary Table 4 was compiled with the characteristic temperatures of the forward and reverse MTs for the samples of the TiNiHf system.

**Figure 8:**
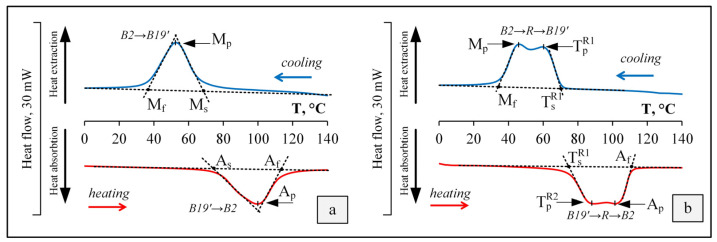
Calorimetric curves of the TiNiHf rods after RF (**a**) and RF + PDA at 550 °C for 2 h (**b**).

The results of the MT study showed that an increase in the Hf content in the initial cast state led to an increase in the finishing temperature of reverse MT A_f_, from 79 °C with an Hf content of 4.4 wt.%, up to 126 °C with 14.8 wt.%. The temperature range of the forward MT also shifted towards higher temperatures with the increase in Hf content. Homogenization annealing at 1000 °C for 1 h led to a noticeable narrowing of the hysteresis of MT due to an increase in the homogeneity of the structural state, and did not lead to the noticeable shift of the temperature range of the reverse MT. The DSC curves of TiNiHf alloy rods after RF and PDA are shown in Figure 8.

Based on the analysis of the obtained calorimetric curves, summary Table 5 was compiled with the characteristic temperatures of the forward and reverse MTs for the samples of the TiNiHf system.

The obtained results showed that RF led to a noticeable increase in the finishing temperature of reverse MT A_f_ as compared to the cast state, from 79 to 113 °C. The application of PDA led to the change in the sequence of the forward and reverse MTs: the transformations proceeded through the intermediate R-phase, while after RF, the transformations proceeded in one stage. The change in the stages of MT can be associated with the influence of static aging processes that develop during annealing and, as a consequence, an increase in the density of structure defects due to the precipitation of the Ti_3_Ni_4_ excess phase (H-phase). This result also showed that the Ni content in the sample was higher than was suggested (more than 50.2 at.%). In this case, there was no noticeable change in the characteristic temperatures of the forward and reverse MTs after PDA.

### 3.4. Temperature Ranges of Martensitic Transformations

**Figure 9:**
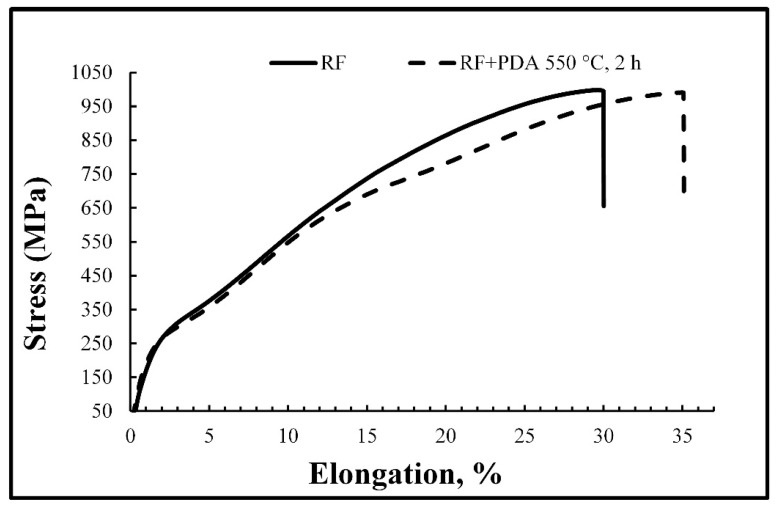
Representative stress–strain diagrams of TiNiHf rods after RF and RF + PDA at 550 °C for 2 h.

The mechanical properties of TiNiHf SMA rods are shown in Table 6. Representative stress–strain diagrams are shown in Figure 9.

The obtained rods showed high strength characteristics: σ_y_ = 800 MPa, σ_B_ = 1000 MPa after RF and σ_y_ = 840 MPa, σ_B_ = 990 MPa after PDA at 550 °C for 2 h, and, simultaneously, sufficiently high ductility: δ = 24% after RF and 29% after PDA. Results of the Vickers hardness test revealed that after RF, the value of hardness was 210 HV. The application of PDA did not noticeably change the hardness value.

The results of determining the temperature range of shape recovery (TRSR) after strain inducing are shown in Table 7. A comparison of the temperature range of the reverse martensitic transformation obtained by DSC (Table 5) and TRSR (Table 7) reveals that strain inducing led to an increase in the temperature of starting and finishing the reverse martensitic transformation.

Based on the presented results, it can be concluded that after RF, the finishing temperature of shape recovery after 2% of induced strain was 125 °C. A induced strain of 6% led to an increase in the A_f_ temperature to 155 °C. The SRR, after 6% of induced strain, was 83%, as the value of total completely recoverable strain was 5%. PDA at a temperature of 550 °C for 2 h allowed increasing A_f_ to 180 °C, while the SRR decreased to 67%. The difference between RF and RF + PDA states consisted in the development of the softening processes during PDA and corresponding increase in TRSR. PDA at a temperature of 1000 ° C for 1 h did not lead to a noticeable change in the A_f_ temperature in comparison to the hot-forged state, while the SRR also decreased to 67%.

## 4. Conclusions

The study of the possibility of the production of long-length rods from TiNiHf alloys, with a lowered Hf content and a high-temperature shape memory effect in the range of 120–160 °C, was performed in the present work. The following conclusions can be drawn:

1. Initial ingots with 1.5, 3.0 and 5.0 at.% Hf were successfully obtained by electron beam melting from Ti-Ni billets and pure Hf wire, used as raw materials.
2. Ingots with 3.0 and 5.0 at.% Hf demonstrated insufficient technological plasticity, presumably because of the excess precipitation of (Ti,Hf)_2_Ni-type particles with the decrease in Ni content. The premature destruction of ingots during the process of rotary forging does not allow obtaining high-quality long-length rods.
3. A good-quality rod with a diameter of 3.5 mm and a length of 870 mm was obtained from the ingot with 1.5 at.% Hf. The obtained TiNiHf rod had relatively high values of mechanical properties: a dislocation yield stress of 800 MPa, ultimate tensile strength of 1000 MPa, and elongation to fracture of 24%.
4. The obtained rods provided the required values of functional properties: a completely recoverable strain of 5%, and a finishing temperature of shape recovery after 2% of induced strain of 125 °C in the as-forged state and of 155 °C after post-deformation annealing at 550 °C for 2 h.

## Figures and Tables

**Table 1:** Chemical composition of the TiNi SMA rod with a diameter of 12 mm, used as a charge material for melting of TiNiHf initial ingots.

Ni, wt.%	Ti, wt.%	Impurities, wt.%				
		C	O	N	H	Other
55.15	Balance	0.035	0.030	0.002	0.001	<0.1

**Table 2:** Chemical composition of the hafnium wire with a diameter of 2 mm, used as a charge material for melting of TiNiHf initial ingots.

Hf, wt.%	Impurities, wt.%						
	C	O	N	H	Si	Fe	Zr
Balance	0.008	0.020	<0.005	0.001	<0.005	0.030	0.58

**Table 3:** Chemical composition of the melted TiNiHf ingots.

No.	Dimensions, mm	Weight, g	Composition (without Impurities)					
			wt.%			at.%		
			Ti	Ni	Hf	Ti	Ni	Hf
1	11 × 16 × 162	102	42.9	52.7	4.4	49.0	49.5	1.5
2	10 × 15 × 230	160	40.4	49.6	10.0	48.5	48.5	3.0
3	11 × 17 × 200	168	38.2	47.0	14.8	47.5	47.5	5.0

**Table 4:** Characteristic temperatures of forward and reverse MTs of TiNiHf initial ingots in as-cast state and after annealing at 1000 °C for 1 h.

Hf Content	Annealing	M_s_, °C	$M^{p},$ °C	M_f_, °C	A_s_, °C	$A^{p},$ °C	A_f_, °C	A_s_ − A_f_, °C	M_s_ − M_f_, °C
1.5 at.% Hf	-	54	28	−12	32	62	79	46	66
1.5 at.% Hf	+1000 °C, 1 h	45	38	27	52	70	79	26	18
3.0 at.% Hf	-	71	60	40	71	107	114	43	31
3.0 at.% Hf	+1000 °C, 1 h	78	70	53	99	117	121	22	25
5.0 at.% Hf	-	71	55	35	76	110	123	47	36
5.0 at.% Hf	+1000 °C, 1 h	80	62	47	86	120	126	40	33

**Table 5:** Characteristic temperatures of forward and reverse MTs of TiNiHf rods after RF and RF + PDA at 550 °C for 2 h.

TMT	$T_{s}^{R1},\;{}^{\circ}C$	$T_{p}^{R1},\;{}^{\circ}C$	$T_{f}^{R1},\;{}^{\circ}C$	M_s_, °C	$M^{p},\;{}^{\circ}C$	M_f_, °C	$T_{s}^{R2},\;{}^{\circ}C$	$T_{p}^{R2},\;{}^{\circ}C$	$T_{f}^{R2},\;{}^{\circ}C$	A_s_, °C	$A^{p},\;{}^{\circ}C$	A_f_, °C	$M_{s}/T_{s}^{R1}-A_{f},\;{}^{\circ}C$	$M_{s}/T_{s}^{R2}$ − M_f_, °C
RF	-	-	-	67	54	36	-	-	-	76	99	113	37	31
RF + 550 °C, 2 h	70	57	-	-	45	35	75	89	-	-	102	116	25	44

**Table 6:** Mechanical properties of TiNiHf rods after RF and RF + PDA at 550 °C for 2 h.

TMT	σ_cr_, MPa	σ_y_, MPa	Δσ, MPa	σ_B_, MPa	δ, %	HV
RF	214	800	586	1000	24	210
RF + 550 °C, 2 h	202	840	638	990	29	215

**Table 7:** Total completely recoverable strain and TRSR of TiNiHf rods after RF, RF + PDA at 550 °C for 2 h, and RF + PDA at 1000 °C for 1 h.

TMT	Induced Strain, %	Total Completely Recoverable Strain, %	SRR, %	TRSR, °C
RF	2.0	2.0	100	65–125
RF	6.0	5.0	83	100–155
RF + 550 °C, 2 h	2.0	2.0	100	110–155
RF + 550 °C, 2 h	6.0	4.0	67	70–180
RF + 1000 °C, 1 h	6.0	4.0	67	120–155

## Data Availability

Not applicable.
